# Suppression of Enhanced Physiological Tremor via Stochastic Noise: Initial Observations

**DOI:** 10.1371/journal.pone.0112782

**Published:** 2014-11-14

**Authors:** Carlos Trenado, Florian Amtage, Frank Huethe, Jürgen Schulte-Mönting, Ignacio Mendez-Balbuena, Stuart N. Baker, Mark Baker, Marie-Claude Hepp-Reymond, Elias Manjarrez, Rumyana Kristeva

**Affiliations:** 1 Department of Neurology and Neurophysiology, University Freiburg, Freiburg, Germany; 2 Institute for Medical Biometry and Medical Informatics, University Freiburg, Freiburg, Germany; 3 Facultad de Psicología, Benemérita Universidad Autónoma de Puebla, Puebla, México; 4 Institute of Neuroscience, Newcastle University, Newcastle upon Tyne, United Kingdom; 5 Institute of Neuroinformatics, University of Zürich and ETH Zürich, Zurich, Switzerland; 6 Instituto de Fisiología, Benemérita Universidad Autónoma de Puebla, Puebla, México; University Medical Center Goettingen, Germany

## Abstract

Enhanced physiological tremor is a disabling condition that arises because of unstable interactions between central tremor generators and the biomechanics of the spinal stretch reflex. Previous work has shown that peripheral input may push the tremor-related spinal and cortical systems closer to anti-phase firing, potentially leading to a reduction in tremor through phase cancellation. The aim of the present study was to investigate whether peripherally applied mechanical stochastic noise can attenuate enhanced physiological tremor and improve motor performance. Eight subjects with enhanced physiological tremor performed a visuomotor task requiring the right index finger to compensate a static force generated by a manipulandum to which Gaussian noise (3–35 Hz) was applied. The finger position was displayed on-line on a monitor as a small white dot which the subjects had to maintain in the center of a larger green circle. Electromyogram (EMG) from the active hand muscles and finger position were recorded. Performance was measured by the mean absolute deviation of the white dot from the zero position. Tremor was identified by the acceleration in the frequency range 7–12 Hz. Two different conditions were compared: with and without superimposed noise at optimal amplitude (determined at the beginning of the experiment). The application of optimum noise reduced tremor (accelerometric amplitude and EMG activity) and improved the motor performance (reduced mean absolute deviation from zero). These data provide the first evidence of a significant reduction of enhanced physiological tremor in the human sensorimotor system due to application of external stochastic noise.

## Introduction

Enhanced physiological tremor (EPT) is an intrusive and disabling form of physiological tremor which emerges during periods of muscular fatigue, fear or excitement and in certain pathological conditions, for example, hyperthyroidism [1]. EPT is characterized as a visible postural and kinetic tremor [6]. EPT is important, as it imposes a strong limitation to fine finger movement precision in both healthy subjects and patients with neurological conditions.

In 1985 Sanes [35] reported an absence of EPT in patients without muscle or cutaneous afferents, leading to the idea that EPT could be produced by several factors including unknown properties of cutaneous receptors, mechanical filtering properties of limb muscles, and fluctuations in motoneuron electrical activity, and synchronization by feedback of the muscle spindles. In agreement with these ideas, electrophysiological studies show that EPT is characterized by a mechanical component activated by the spinal stretch reflex as well as a central component consisting of a neural oscillator at 8–12 Hz [6].

Although there is some understanding of these mechanisms, little is known about practical procedures to reduce EPT. Most treatments are pharmacological or surgical and are targeted at essential tremor [3], [18]–[20], physiological tremor [4], [5], [7], [8], and Parkinsonian tremor [11], [23]. Other methods to reduce tremor employ robotics-assisted technologies [24], [32], [34], [40] or rely on precise characterization of the tremor properties, allowing tremor modulation via wearable functional electrical stimulation devises [12], [13], [29], [33].

Recently, Williams et al [43] found in monkeys that several supraspinal centers (primary motor cortex (M1), the pontomedullary reticular formation and deep cerebellar nuclei) exhibit oscillatory activity at tremor frequency in anti-phase with oscillations in the spinal cord. Convergence of supraspinal and spinal inputs at the motoneuron level could produce tremor cancellation and improve movement precision. In support of this suggestion, Koželj and Baker [22] demonstrated in monkeys that different phase delays of peripheral input promote anti-phase firing between spinal cord and cortex. Recently we reported improved sensorimotor performance after the application of external mechanical noise, a phenomenon known as ‘stochastic resonance’ [30], [38], [39]. We speculated that this improved sensorimotor performance may occur concomitantly with tremor reduction, because artificially boosting the strength of peripheral input biases the spinal and cortical systems closer to anti-phase firing, and lead to a reduction in tremor.

In this study we investigated performance of a visuomotor task in patients with EPT, comparing performance and tremor in conditions with and without superimposed noise. The application of optimum noise reduced tremor and improved the motor performance.

Our findings could lead to the development of new devices based on mechanical stochastic noise to reduce tremor for clinical benefit.

## Materials and Methods

### Subjects

Eight patients with enhanced physiological tremor (2 females, mean age 38±17 years) took part in the study. All were recruited based on routine clinical tremor analysis during a consultation in the outpatient neurology department. Table 1 shows patients' characteristics. Handedness was assessed with the Oldfield questionnaire [31]. No patients had previously participated in similar experiments before. Informed written consent was obtained in accordance with the Declaration of Helsinki and all procedures were approved by the Ethics Committee of the Medical Faculty, Freiburg.

**Table 1:** Patients' characteristics.

Subject No	Age [years]	Gender [M/F]	Approx. disease duration [years]	Acceleration Peak frequency [Hz]	Tremor EMG frequency right [Hz]
1	44	M	28	8.0	8.0
2	62	M	5	9.9	9.9
3	20	F	4	10.0	10.0
4	25	M	3	11.7	11.7
5	21	F	3	12.0	9.9
6	25	M	3	11.6	11.8
7	49	M	3	8.0	8.0
8	55	M	3	7.0	8.0

### Experimental paradigm

#### Paradigm

**Figure 1:**
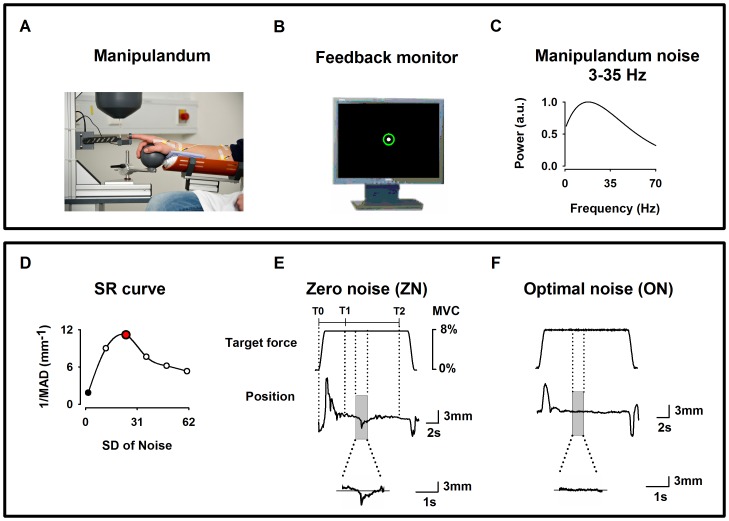
Experimental setup. (**A**) Home-made index finger manipulandum producing a target static force (8% of individual maximum voluntary contraction) on which noise in the frequency bandwidth 3–35 Hz is added. Profile of the target static force in **E** and **F**. EMG from the right first dorsal interosseus (FDI), the right flexor digitorum superficialis (FDS) and the right extensor digitorum communis (EDC) muscles were recorded. (**B**) Visual feedback of the finger position as a solid white dot within a green circle indicating the tolerance for position errors, displayed on a monitor in front of the subject. (**C**) Spectral power of the noise of the manipulandum in arbitrary units (au) for the frequency bandwidths 3–35 Hz). (**D**) Effect of the stochastic resonance (SR) on the motor performance of one subject recorded prior to the experimental session and computed as the inverse of the mean absolute deviation (1/MAD) of the finger position. Note the inverted U-shape like curve. During the experiment only two noise levels were individually chosen, i. e. zero noise (ZN, black filled dots) and optimal noise (ON, red filled dots). (**E, F**) Original signals for target force and finger position (representing the exerted force) for ZN (**E**) and ON (**F**). Transitory phase of the task between markers T0 and T1 and stationary phase between markers T1 and T2. Only data belonging to the stationary part of the force (between T1 and T2) was taken for the analysis. Note in the magnified position traces the better performance for ON than for ZN.

During the experiment, the patient sat in an electrically shielded, dimly lit room. The right arm was supported by a splint and the subject was instructed to place the right hand over a sphere and the index finger in the ring of a custom-made manipulandum (*see* Fig. 1A).

The manipulandum was designed to produce a vertical force in the upward direction on the ring. The subject had to compensate and maintain a target force (Fig. 1E, F) quasi-isometrically by applying force at the level of the metacarpophalangeal joint in the opposite direction (downwards).

#### Force profile

The target force shown in Fig. 1E, F was set at 8% of the maximum voluntary contraction determined for each subject prior to the experiment. We used this low force as motor cortical neurons are most sensitive to forces within a low force range [16], [17]. Papers from the Hepp-Reymond group have shown that M1 neurons have a high slope of the relationship between force and firing rate – 69 Hz/N [41]. Given that tonic firing rates of cortical neurons are typically no larger than 100–150 Hz, this indicates that cortical coding can only represent low forces accurately.

Each trial comprised three phases: a *1 s ramp phase* (rising cosine function to ensure a smooth start) followed by 12 s-period of *static force* (SF), followed by *downward ramp phase* (also cosine function).

#### The manipulandum

The manipulandum used in this study is an improved version of the manipulandum used in [30] and is described in detail in [38].

#### Visual feedback

The feedback of the force exerted by the patient was displayed as the position of a white dot (radius 2 mm) on a 19″ monitor placed 100 cm in front of him/her (Fig. 1B). This white dot moved vertically within a fixed green circle (radius 6 mm including the line thickness of 2 mm) representing the range within which the white dot was allowed to move. When the force was applied to the ring and thus to the right index finger, the subject had to compensate by applying a quasi-isometric force in the opposite direction to maintain the white dot in the middle of the green circle. Thus, the feedback to the subject was a positional one, representing the difference between forces applied by the subject and the force generated by the manipulandum. A finger displacement of 1 mm corresponded to 2.85 mm visual feedback. The allowed tolerance for positional errors was indicated by the green circle. Data segments in which the white point exited the green circle were excluded from further analysis; such segments represented approximately 1% of the total.

#### Experimental conditions

In the present experiment tactile Gaussian noise in the range 3–35 Hz was applied to the manipulandum and thus added to the target force (Fig. 1C).

Prior to the experiment the following tests were performed:

*First*, we defined the maximum voluntary contraction (MVC) individually.

*Second*, the patient performed a few trials to allow familiarization with the task and learn “what” to do and “how” to do it.

During the experimental session, the first seven recording series of five trials each were collected for the zero noise (ZN) condition (35 trials).

To ensure that subjects sustained their attention during the experiment, they had to report after each series of five trials whether the trials were with or without added noise. The subjects were instructed to avoid any other movements and to fix their gaze on the visual feedback during the trials.

After these seven recording series, we defined for each patient the noise level which could be considered as optimum noise (ON). To this end, we made use of a MATLAB customized program that delivered a force at 8% MVC during 110 s and added noise levels in an incremental fashion (10 levels of noise were administered in steps of 10 mN, each one lasting 10 s, with an additional 10 s at the beginning comprising a baseline period, a ramp phase to reach 8% MVC and a period to avoid transitory effects). Immediately after that we calculated the performance as a function of the noise level, i.e. the stochastic resonance (SR) curve. Fig.1D shows an example of an inverted U-like curve, which is the signature of the SR, as a function of noise intensity for a representative patient. We defined the ON as the noise level inducing the best performance, as measured by the smallest absolute deviation from 0 (the biggest value of the inverse of mean absolute deviation (*MAD*). The procedure was repeated five times to ensure reliability of the measures. This objectively defined ON level coincided with the noise level at which the patient reported that his/her tremor was best suppressed.

Seven recording series of five trials each were then collected for the optimum noise (ON) condition thus reaching 35 trials.

To avoid fatigue, rest intervals of 5 to 7 s were included between trials. Rest periods of about 5 min between the series were given to avoid adaptation to the perception of the noise [2].

At the end of the experimental session the subjects had to report whether the noise had helped them to be more precise.

### Recordings

Electromyographic activity (EMG, bandpass 30–500 Hz; sampling rate 2000 Hz was recorded with surface electrodes using a belly-tendon montage from the pars indicis of the right flexor digitorium superficialis (FDS), the right first dorsal interosseus (FDI), and the right extensor digitorum communis (EDC) muscles. Our task requires synergetic co-contraction of these three muscles, which have intermingled cortical representations [27], [28], [36], [37].

The force and displacement of the finger (position, POS) were recorded in parallel with the electrophysiological data (bandpass DC-500 Hz and sampling rate 2000 Hz). Data were stored and analysed off-line.

### Data analysis

#### Calculation of performance

To test the effects of the SR phenomenon on the finger performance, the mean absolute deviation (MAD) was computed on the basis of the formula:
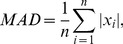
(1)where *x_i_* is the value of finger position (centred on the target position) in response to the applied force at the sampling point *i*. *MAD* measures the deviation amplitude of the white dot relative to the fixed green circle during the application of the force on the manipulandum in the y-direction.

#### Calculation of the acceleration

To test the effects of the SR on the tremor, the finger acceleration was calculated from the second derivative of the position trace (low pass filter 15 Hz), as implemented in the commercial software “Brain Vision 2.0.2” (München, Germany).

#### Calculation of the EMG spectral power (SP)

To test the effect of the noise on the muscle activity, the SP of the EMG was calculated according to the following equation:
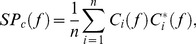
(2)where *C_i_* represents the Fourier transformed EMG channel *c* for a given segment number (*i* = 1,…,*n*) and * denotes the complex conjugate. Rectified EMG was used. We are aware about the debate whether the rectification of EMG as pre-processing step is necessary [10], [26]. However, based on our own experience and the experience of others [42], we use rectification of surface EMG to enhance accuracy of the EMG timing information prior to spectral analysis [9].

Having in mind that the EMG amplitudes cannot be compared between muscles we did a comparison for each muscle individually between both conditions by means of the EMG spectral power.

### Statistical analysis

#### Statistical analysis of the EMG and acceleration spectral power (SP)

To test for statistical difference in acceleration spectral power between ZN and ON, we computed the individual areas under the SP curve, *A_pow_*. The frequency windows for *A_pow_* were 7–12 Hz (tremor range), 15–30 Hz (beta range) and 30–45 Hz (gamma range). To prepare data for statistical analysis, data were logarithmically transformed to yield symmetric distributions according to the formula:
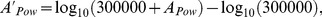
(3)where the *first value* (300000) has been selected to fulfil: a) homogeneity of variance, and b) symmetry of distribution. The *second value* (log_10_(300000)) was defined in such a way that the transformation maps 0 to 0.

#### Statistical analysis to exclude fatigue during the experiment

To exclude fatigue during the experiment, we compared the mean peak frequency of the EMG power spectrum between first and second part of the ZN condition, between first and second part of the ON condition and between ZN and ON conditions. For this purpose we applied two factor (time: first and second part) and noise (ZN and ON) repeated measures ANOVA.

#### Statistical analysis of performance

To account for the inter-subject variability and achieve symmetry of the data distribution, the *MAD* values were also first logarithmically transformed according to the formula
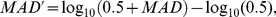
(4)where the *first value* (0.5) has been selected to fulfil: a) homogeneity of variance and b) symmetry of distribution. The *second term* (log_10_(0.5)) was defined in such a way that the transformation maps 0 to 0.

For EMG spectral power, acceleration and performance, if ANOVA factors and interaction effects were significant, we performed paired Wilcoxon tests, where the null hypotheses were that the differences of the means between ZN and ON were zero.

## Results

### Behavioural performance

All eight patients performed the task according to the instructions. None of them reported fatigue or anxiety during the experimental session. All of them reported that their tremor was reduced when noise was applied and their performance was better. The absence of fatigue was supported not only by the subjective report of the patients but as well by the absence of significant difference in the mean peak frequency of the EMG power between first and second part of the ZN condition, between first and second part of the ON condition and between ZN and ON conditions as supported by the non-significant differences for both “noise” and “part” conditions, as well as their interaction. Numerical data for the peak frequency of the EMG power are shown in Table 2.

**Table 2:** EMG mean power frequency.

	ZN (Hz)		ON (Hz)	
Subject No	First half	Second half	First half	Second half
1	5,85	9,76	5,85	7,81
2	9,76	13,67	13,67	11,71
3	9,76	9,76	9,76	9,76
4	11,71	13,67	1171	11,71
5	13,67	11,71	13,67	13,66
6	11,71	11,71	11,71	11,71
7	7,81	7,81	7,81	7,81
8	7,81	7,81	7,81	7,81
Mean	9,76	10,73	10,73	10,735
SD	2,5	2,3	2,9	2,2

All patients reported that tremor was reduced after the experiment. Specifically, one reported that the after-effect of the stimulation lasted up to fifteen hours, two up to eight hours, one up top six hours, another one up to four hours, and three reported that the effect lasted up to three hours.

**Figure 2:**
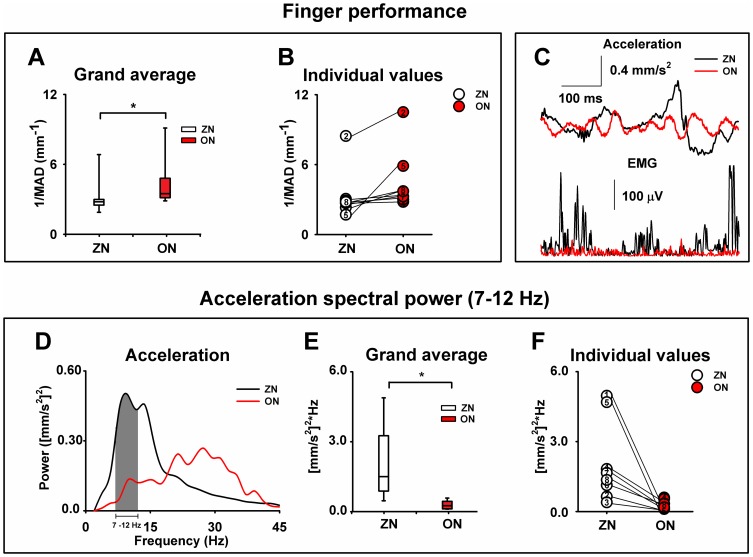
Motor performance (1/MAD), raw acceleration and raw rectified EMG signals and spectral power of acceleration for zero noise (ZN, black) and optimum noise (ON, red). **Upper panel**: Grand average of the inverse of the mean absolute deviation (1/MAD) of the finger position in (**A**) and the individual (1/MAD) values for each subject in (**B**). Note the stochastic resonance (SR) effect with better performance for ON. (**C**) Raw acceleration signal and raw EMG signal showing the tremor reduction. **Lower panel**: Grand average of the accelerometric power spectra for all patients in (**D**) and (**E**) with either ZN or ON and individual values in (**F**). Note the great reduction of the accelerometric spectral power as an effect of ON.

Fig. 2 shows the better performance when optimum noise was applied. The mean absolute deviation (MAD) values were significantly smaller in the ON than in the ZN condition as seen from the grand average of the performance (Fig. 2A) and from the individual MAD values (Fig. 2B) (p = 0.008, Wilcoxon paired test; n = 8 throughout the whole text).

Fig. 2C depicts raw acceleration and raw rectified EMG signals showing the tremor reduction in both, acceleration and EMG. Fig. 2D shows the grand average of the acceleration power spectrum for ZN and ON which shows tremor suppression in the 7–12 Hz frequency range. This can be better appreciated from the area under the curve in the tremor frequency band for both averaged (Fig. 2E; p = 0.0078, Wilcoxon paired test) and individual data (Fig. 2F). Figure 2D also shows stronger SP between 15 and 35 Hz during the ON condition, although the difference with ZN was not significant. This reflects most probably some mechanical effect from the added noise, which included power in the beta band.

To investigate whether the tremor suppression and performance improvement were correlated we calculated Spearman correlation coefficients. A non-significant negative correlation between both variables was found (data not shown).

### EMG spectral power

**Figure 3:**
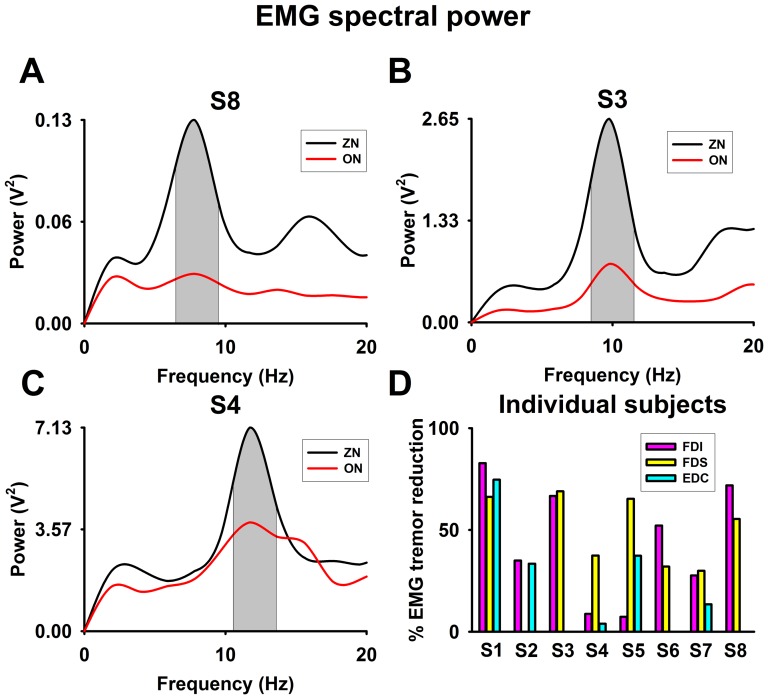
EMG spectral power for zero noise (ZN, black) and optimum noise (ON, red). (**A–C**): Power spectra for FDI muscle activity for subject 8 and for FDS muscle activity for subjects 3 and 4 with ZN and ON, highlighting the tremor frequency. (**D**) For all eight subjects, effect of ON on the EMG power spectra (i.e. % of EMG tremor reduction) of FDI, FDS, EDC muscles (at the individual tremor frequency).

Figure 3 A–C depicts example frequency power spectra from the FDI muscles for subject 8 and from the FDS muscle activity for subjects 3 and 4. The reduction in the tremor frequency band is clearly visible. Figure 3D illustrates for individual data of the EMG spectral power reduction for all muscles at the tremor frequency, with reductions of 44±29% for the FDI, 44±24% for the FDS and 20±27% for the EDC (mean ± SD).

Correlation analysis between the reduction in EMG tremor power and in accelerometric spectral power revealed a positive but non-significant Spearman correlation (FDS, r = 0.43, p = 0.29; EDC, r = 0.45, p = 0.27).

Our results provide support for a significant effect of noise on the FDS and FDI muscles in the suppression of tremor during the performance of the visuomotor task.

## Discussion

This study shows for the first time that optimum Gaussian noise has a great impact reducing the amplitude of EPT, as well as improving performance in a complex sensorimotor task. This study was planned as a pilot trial to estimate the possible effects of optimum noise on tremor activity. Even with a small number of patients we saw significant effects on both tremor reduction and accuracy of performance due to optimum noise. We exclude a placebo effect since all eight subjects reported that their tremor was maximally reduced with the application of ON, i.e. an optimal level of stochastic noise, and less at both larger and smaller noise levels. Moreover, the subjects did not know at what time the optimum noise level was applied. Tremor reduction was detectable by both accelerometric analysis and by EMG recordings. The lack of significant correlation between the tremor power change and EMG level change means that tremor did not scale with motor output. Furthermore, of considerable interest was that tremor alleviation outlasted the period of stimulation by up to fifteen hours. In future experiments we will make in addition objective evaluation of the tremor after the experiment over a longer period of time.

We observed that the percentage of EMG tremor reduction (Fig. 3D) was higher for flexor muscles (FDI and FDS) than for extensor muscle (EDC). This difference could be explained by the nature of the task performed, that mainly activates the flexor muscles (FDI and FDS).

Since optimum noise is associated with a decrease of both the acceleration spectral power (Fig. 2E) and the percent of EMG spectral power (Fig. 3D), we speculate that optimum noise has a direct influence on the central (brain/brainstem/cerebellum) oscillatory component of tremor. This is very noteworthy since this influence arises from a manipulation of peripheral input to the CNS, showing that afferent signals are able to modulate the tremor generating network.

All patients showed a better performance with optimum noise as found by us also in healthy subjects [30], [38]. It is tempting to assume that this is based on tremor reduction. However, we found no significant correlation between performance improvement and tremor reduction. Therefore, other mechanisms besides tremor reduction are likely also to play a role in patients with EPT. One possibility is better sensorimotor integration due to enhanced sensitivity of cutaneous receptors and increased afferent input, leading to a stronger cortico-muscular synchrony [30], [38], [39].

Little is known about the role and location of the primary oscillator in EPT. To our knowledge the first report on the possible origin of EPT is attributed to Köster and colleagues [21], who found evidence of a bilateral oscillator for EPT linking both hemispheres. They suggested that EPT is produced by transcortical transmission of cortical oscillators impinging on the corticospinal tract: This possibility was supported by earlier studies: The first study was reported by Sanes in 1985 [35], who described an absence of EPT in patients without muscle or cutaneous afferents. The second study was reported by Logigian et al. [25], who demonstrated that the greater the amplitude of EPT the larger was the amount of motor unit synchronization. However, in a study by Hellwig et al. [15] cortico-muscular coherence could not be detected in three patients with EPT, most probably due to the low tremor amplitude or a poor signal to noise ratio. The latter possibility is supported by other study in tremor patients with idiopathic Parkinson disease in which highly significant coherence at the tremor frequency or at its first harmonic was found between the tremor EMG and contralateral EEG channels [14].

In conclusion, we provide the first evidence of a significant reduction of EPT due to the application of external stochastic noise. This finding could help to understand the origin of EPT and to yield clinical benefit for patients by developing new devices based on mechanical stochastic noise.
